# Preoperative Prediction of Lymph Node Metastasis in Colorectal Cancer with Deep Learning

**DOI:** 10.34133/2022/9860179

**Published:** 2022-03-16

**Authors:** Hailing Liu, Yu Zhao, Fan Yang, Xiaoying Lou, Feng Wu, Hang Li, Xiaohan Xing, Tingying Peng, Bjoern Menze, Junzhou Huang, Shujun Zhang, Anjia Han, Jianhua Yao, Xinjuan Fan

**Affiliations:** ^1^Department of Pathology, The Sixth Affiliated Hospital of Sun Yat-sen University, Guangzhou 510655, China; ^2^AI Lab, Tencent, Shenzhen 518057China; ^3^Department of Computer Science, Technical University of Munich, Munich 85748, Germany; ^4^Department of Computer Science, Xiamen University, Xiamen 361005, China; ^5^Department of Electronic Engineering, The Chinese University of Hong Kong, Hong Kong 999077, China; ^6^Institute of Computational Biology, Helmholtz Zentrum München, Neuherberg 85764, Germany; ^7^Helmholtz AI, Helmholtz Zentrum München, Neuherberg 85764, Germany; ^8^Department of Quantitative Biomedicine, University of Zurich, Zurich 8091, Switzerland; ^9^Department of Pathology, The Fourth Hospital of Harbin Medical University, Harbin 150001, China; ^10^Department of Pathology, The First Affiliated Hospital of Sun Yat-sen University, Guangzhou 510080, China

## Abstract

*Objective*. To develop an artificial intelligence method predicting lymph node metastasis (LNM) for patients with colorectal cancer (CRC). *Impact Statement*. A novel interpretable multimodal AI-based method to predict LNM for CRC patients by integrating information of pathological images and serum tumor-specific biomarkers. *Introduction*. Preoperative diagnosis of LNM is essential in treatment planning for CRC patients. Existing radiology imaging and genomic tests approaches are either unreliable or too costly. *Methods*. A total of 1338 patients were recruited, where 1128 patients from one centre were included as the discovery cohort and 210 patients from other two centres were involved as the external validation cohort. We developed a Multimodal Multiple Instance Learning (MMIL) model to learn latent features from pathological images and then jointly integrated the clinical biomarker features for predicting LNM status. The heatmaps of the obtained MMIL model were generated for model interpretation. *Results*. The MMIL model outperformed preoperative radiology-imaging diagnosis and yielded high area under the curve (AUCs) of 0.926, 0.878, 0.809, and 0.857 for patients with stage T1, T2, T3, and T4 CRC, on the discovery cohort. On the external cohort, it obtained AUCs of 0.855, 0.832, 0.691, and 0.792, respectively (T1-T4), which indicates its prediction accuracy and potential adaptability among multiple centres. *Conclusion*. The MMIL model showed the potential in the early diagnosis of LNM by referring to pathological images and tumor-specific biomarkers, which is easily accessed in different institutes. We revealed the histomorphologic features determining the LNM prediction indicating the model ability to learn informative latent features.

## 1. Introduction

Colorectal cancer (CRC) remains the third most common malignancy and is a leading cause of cancer-related mortality in the world, despite the improvement in the overall outcomes due to the development of new cancer treatments and management [1]. Preoperative neoadjuvant chemoradiotherapy (nCRT) followed by total mesorectal excision (TME) significantly reduces local recurrence and shows favourable prognosis; thus, it has become the standard therapeutic regimen for locally advanced rectal cancer (clinically staged as N1-2) [2, 3]. Moreover, for patients with node negative T1 lesions (N0 staging), endoscopic submucosal excision is recommended, and no additional surgery is required [4]. Thus, preoperative clinical nodal staging is critical to determine the treatment strategy. However, the prediction of lymph node metastasis (LNM) status before surgery remains challenging for CRC.

Preoperative imaging such as computed tomography (CT) and magnetic resonance imaging (MRI) is currently the most common approach for assessing LNM, which is considered the gold standard for node staging. Node size, border, shape, and intensity are the main criteria for evaluating whether the node has metastatic lesions [5]. Kim et al. found that the accuracy rates of MRI and CT for LMN of rectal cancer were 63% and 56.5%, respectively [6]. The criteria of diagnosing metastasis are mainly based on the size and shape of lymph nodes, where micrometastasis may be filtered out. The guideline varies from institution to institution. For instance, others employ size criteria with cutoff values for nodal positivity that range from 3 to 10 mm [7]. A meta-analysis reported that the sensitivities and specificities of these imaging tests were 53-88% and 60-97%, respectively, and the area under curve (AUC) ranged from 0.66 to 0.79 [8]. In addition to imaging approaches, molecular tests have been reported for LNM prediction. Ozawa et al. identified 5 microRNAs (MIR32, MIR181B, MIR193B, MIR195, and MIR411) and showed that these microRNAs were significantly changed in T1 and T2 CRC patients, and the AUC value was 0.77 for biopsy serum samples [9]. However, the high testing expenses and instability of microRNAs in serum hinder their widespread application.

Deep learning, as one of the most advanced machine-learning methodologies, has recently shown record-breaking performance in many challenging medical tasks, including disease diagnosis, treatment, and prognosis [10, 11]. Different from conventional machine-learning methods that mainly rely on handcrafted features, deep learning has the advantage of being able to learn latent features automatically and effectively [10]. Recent studies have shown the potential success of deep learning in achieving competitive and even superior performance compared to human experts on multiple tasks in medical image analysis. For example, Esteva et al. demonstrated that the classification of skin lesions using a single deep convolutional neural network (CNN) achieved a level of diagnostic capability comparable to that of expert dermalogists [12]. Similarly, Hannun et al. proposed an end-to-end deep learning method that could achieve cardiologist-level arrhythmia detection and classification using ambulatory electrocardiograms [13]. Recently, deep learning has also shown its potential in digital pathology for tasks such as histopathology diagnosis [14], prognosis [15], gene mutation [16], and the origin prediction for cancers of unknown primary [17]. Although deep learning is rapidly advancing, to the best of our knowledge, it has not been applied in the prediction of LNM from CRC. Besides, most current deep learning-based methods for pathology analysis employ only a single data modality (i.e., histopathological images) [14–18]; however, the combination of multiple complementary data modalities showed superiority when addressing biomedical challenges [19].

In this study, we developed a Multimodal Multiple Instance Learning (MMIL) model based on the deep neural network for predicting LNM. We integrated the information of both the blood biomarker alterations in the serum and the tumor microenvironment in histopathological images into the MMIL model to predicate the LNM status of patients with colorectal cancer. Moreover, we explored and visualized the deep learning features that are most salient to LNM prediction to provide clinicians with an intuitive interpretation of our MMIL model, improving the model transparency and interpretability.

## 2. Results

### 2.1. Study Design and Methodological Development of MMIL

**Figure 1:**
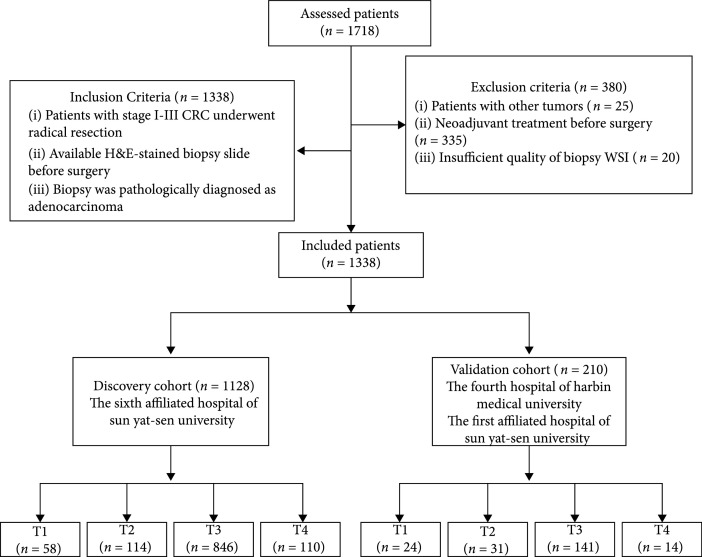
STARD diagram of participants in the study.

**Figure 2:**
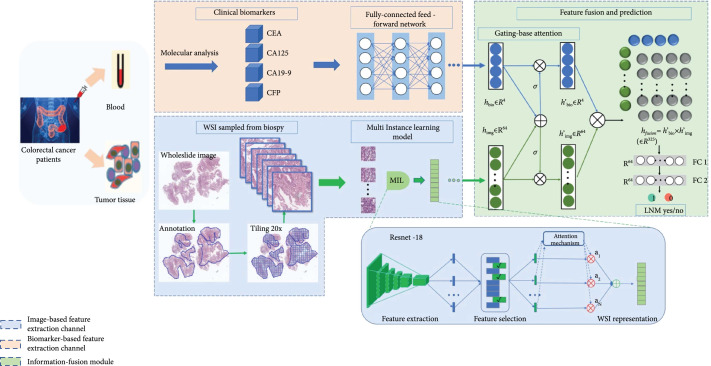
The overall framework of the proposed Multimodal Multiple Instance Learning (MMIL) AI system for predicting lymph node metastasis (LNM). Our system is composed of two feature extraction channels and an information fusion module, i.e., an image-based feature extraction channel that generates a representation of histopathological images via multiple instance learning (MIL), a biomarker-based feature extraction channel that generates a representation of serum tumor-specific biomarkers based on a fully connected network, which are followed by an information fusion module to integrate the obtained features from both channels via a gating-based attention mechanism as well as tensor fusion and to perform the final classification. The MIL method in the image-based feature extraction channel consists of instance-level feature extraction, instance-level feature selection, and bag-level representation generation. ResNet-18 works as an instance-level feature extractor. The feature selection procedure selects discriminative instance-level features. The attention-based deep multiple instance learning model is used to synthesize instance-level features and generate bag representations.

This is a retrospective, multicentre study that recruited CRC patients from three hospitals specializing in gastrointestinal disease in China [the Sixth Affiliated Hospital of Sun Yat-sen University (SYSU6), the First Affiliated Hospital of Sun Yat-sen University (SYSU1), and the Fourth Hospital of Harbin Medical University (HMU4)]. A total of 1338 CRC patients with different T stages (T1 to T4) were recruited and divided into two cohorts: the discovery cohort, including 1128 patients (T1: 58, T2: 114, T3: 846, and T4: 110) from SYSU6; and the external validation cohort, composed of 210 patients (T1: 24, T2: 31, T3: 141, and T4: 14) from SYSU1 and HMU4. The patient inclusion and exclusion criteria are illustrated in Figure 1. The discovery cohort was used to develop and evaluate the MMIL model (Figure 2), and external validation was applied to evaluate its generalization performance. Based on the tumor-node-metastasis (TNM) staging information, the patients were categorized into two groups: without LNM (patients with N0 stage, denoted as LNM-) and with LNM (patients with N1 and N2, denoted as LNM+). The N stage was pathologically diagnosed after radical TME surgery. A more detailed information in the study is summarized in Table 1.

**Table 1:** Clinical features of colorectal cancer (CRC) patients in different T stages.

	T1 stage			T2 stage			T3 stage			T4 stage		
	LNM+	LNM-	P value	LNM+	LNM-	P value	LNM+	LNM-	P value	LNM+	LNM-	P value
*Discovery cohort*												
Number of patients	9	49	/	24	90	/	344	502	/	63	47	/
Sex (male, female)	4, 5	31, 18	0.3728	13, 11	45, 45	0.7545	215, 129	316, 186	0.9950	38, 25	27, 20	0.9614
Age at biopsy (years)	53.2 (13.6)	63.2 (10.3)	0.0166	64.2 (12.4)	64.2 (12.5)	0.9778	58.8 (12.4)	60.2 (12.8)	0.1007	60.0 (12.9)	60.8 (14.3)	0.5659
Location	0, 0, 0, 4, 5	5, 3, 16, 7, 18	0.0166	3, 0, 7, 3, 11	7, 8, 25, 12, 38	0.5135	73, 17, 137, 75, 42	124, 50, 172, 103, 53	0.2778	19, 4, 18, 20, 2	7, 7, 21, 11, 1	0.2485
Histologic grade	4, 5, 0	11, 37, 1	0.2734	9, 14, 1	42, 48, 0	0.3926	107, 204, 33	230, 259, 13	<0.0001	18, 38, 7	15, 29, 3	0.5700
CEA (ng/mL)	2.13 (1.57−2603.00)	2.28 (0.72−10.30)	0.4852	3.21 (0.85−9.28)	2.61 (0.50−1763.15)	0.9584	5.06 (0.50−6039.37)	3.45 (0.50−5389.88)	0.0104	11.49 (0.52−1600.15)	7.85 (0.75−5235.32)	0.7649
CA125 (U/mL)	10.70 (5.10−52.00)	11.20 (3.60−29.80)	0.6832	9.80 (6.00−101.90)	11.20 (4.20−161.50)	0.5758	11.90 (2.00−1493.80)	11.10 (2.00−194.60)	0.0194	16.60 (3.30-1777.70)	17.20 (3.30−455.70)	0.8090
CA19-9 (U/mL)	11.72 (2.00−10865.78)	6.42 (2.00−74.48)	0.2013	2.81 (0.75−42.61)	6.15 (2.00−167.79)	0.6716	12.11 (1.20−120000.00)	8.13 (1.20−66336.20)	<0.0001	21.66 (2.00−120000.00)	13.94 (2.00−3785.11)	0.4684
AFP (ng/mL)	1.80 (0.91−3.74)	2.77 (1.28−10.61)	0.037	2.72 (0.75−9.40)	2.70 (0.75−8.20)	0.1245	2.44 (0.84−9.82)	2.48 (0.72−17.53)	0.5292	2.39 (0.91−6.00)	2.28 (0.78−7.90)	0.5043
*External validation cohort*												
Number of patients	5	19	/	5	26	/	54	87	/	8	6	/
Sex (male, female)	4, 1	13, 6	0.6958	1, 4	13, 13	0.2950	31, 23	60, 27	0.2495	5, 3	3, 3	0.3662
Age at biopsy (years)	65.4 (11.9)	64.2 (12.3)	0.8590	62.6 (14.0)	61.4 (13.3)	0.8509	66.9 (10.3)	66.9 (9.2)	0.9949	62.0 (10.2)	60.2 (13.1)	0.5168
CEA (ng/mL)	4.67 (2.03−5.70)	2.10 (0.79−6.20)	0.4554	1.84 (0.28−2.08)	2.12 (0.32−50.51)	0.1326	5.02 (0.43−96.39)	3.85 (0.52−166.50)	0.8106	8.90 (1.47−131.20)	3.84 (1.45−169.30)	0.7963
CA125 (U/mL)	9.00 (4.30−11.10)	10.35 (3.60−41.90)	0.4994	12.44 (8.57−13.68)	10.31 (5.00−25.63)	0.5912	11.10 (4.67−70.75)	11.72 (4.88−183.40)	0.4683	11.28 (11.02−11.53)	10.68 (10.33−57.48)	0.7963
CA19-9 (U/mL)	4.23 (2.00−12.96)	7.99 (2.00−98.96)	0.6188	12.28 (8.39−26.25)	11.07 (2.79−24.98)	0.5547	16.21 (1.05−324.90)	13.62 (1.76−189.40)	0.9560	8.92 (0.93−34.23)	28.46 (4.39−104.40)	0.3662
AFP (ng/mL)	2.15 (1.94−5.43)	2.53 (1.15−111.28)	0.7491	2.63 (1.47−4.33)	3.00 (1.20−6.36)	0.9145	2.59 (1.01−11.95)	2.89 (0.64−117.60)	0.4327	2.56 (1.23−3.49)	3.98 (1.35−5.51)	0.8973

Values of age at biopsy are mean (SD). Values of biomarkers (CEA, CA125, CA19-9, and AFP) are median (minimum-maximum). Values of location are right-sided colon cancer, transverse colon cancer, left-sided colon cancer, sigmoid colon cancer, and rectal cancer. Values of histologic grade are G1: well differentiated, G2: moderately differentiated, G3: poorly differentiated.

### 2.2. Performance Evaluation in the Discovery Cohort

**Figure 3:**
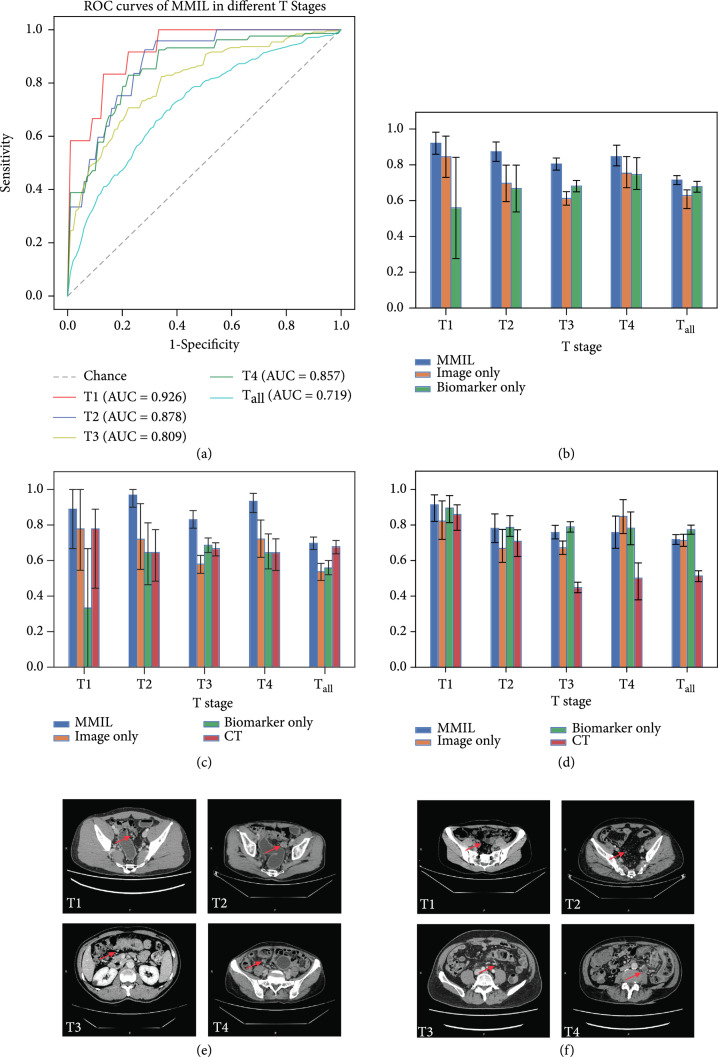
Performance evaluation of LNM prediction by different methods evaluated in the discovery cohort. (a) ROC curves of MMIL in different stages. (b) Area under curves of different methods. (c) Sensitivities obtained by different methods. (d) Specificities obtained by different methods. (e) CT-testing positive LNM were predicted negative using the MMIL model and finally confirmed as negative. (f) CT-testing negative LNM were predicted positive using the MMIL model and finally confirmed as positive. The detailed numbers of (a–c) are given in Supplemental Table 1. Abbreviations: ROC = receiver operating characteristic; AUC = area under the curve; MMIL: multimodal multiple instance learning.

The cross-validation procedure (6-fold for T1, T2, and T4, as well as 10-fold for T3 and T1) was used to proof the concept and evaluate the MMIL model on the discovery cohort. The MMIL model achieved LNM prediction with AUCs of 0.926 (95% CI: 0.864-0.988), 0.878 (95% CI: 0.824-0.933), 0.809 (95% CI: 0.775-0.843), and 0.857 (95% CI: 0.799-0.915) for stage T1, T2, T3, and T4, respectively (Figures 3(a) and 3(b), Supplemental Table 1). Furthermore, we also evaluated the performance of the MMIL method for all patients mixed with different T stages (denoted as T_all_), and the model showed an average AUC of 0.719 (95% CI: 0.694-0.744) (Figures 3(a) and 3(b)).

Our results showed that MMIL had superior predictive value compared with CT-based diagnosis. Taken T1 stage for example, the sensitivity and specificity (88.9% and 91.1%, respectively) of the MMIL model were higher compared to CT-based diagnosis (77.8% and 85.7%, respectively, Figures 3(c)–3(f), Supplemental Table 1). Similar results were found in the other T stages, MMIL outperformed CT-based diagnosis by at least 7.3% in the sensitivity/specificity of patients with stage T2, T3, and T4, and the performance difference ranged from 7.3% to 32.3% (Figures 3(c)–3(f)).

To evaluate the contribution of each data modality, we employed the multiple instance learning network individually using the single image-based feature extraction channel to evaluate the performance of leveraging histopathological images only. Meanwhile, Extreme Gradient Boosting (XGBoost) [20] was employed to combine four serum tumor-specific biomarkers (CEA, CA125, CA19-9, and AFP) to evaluate the performance of leveraging blood biomarker only. The performance comparison between our proposed MMIL method, using histopathological image only and using blood biomarkers only, illustrated the benefit of combining multimodality data in the LNM prediction task. To be specific, the MMIL achieved 0.076 (T1), 0.177 (T2), 0.193 (T3), 0.094 (T4), and 0.087 (T_all_) higher average AUC than the case only relying on the imaging modality (P value: T1 0.261, T2 0.015, T3 $1.554\times 10^{-5}$, T4 $9.443\times 10^{-6}$, T_all_$1.145\times 10^{-6}$). Meanwhile, it also achieved 0.363 (T1), 0.206 (T2), 0.124 (T3), 0.101 (T4), and 0.037 (T_all_) higher average AUC compared to leveraging tumor biomarker only (P value: T1 0.005, T2 $2.548\times 10^{-4}$, T3 $4.258\times 10^{-4}$, T4 0.005, and T_all_ 0.002, Figure 3(b), Supplemental Table 1). For most T stages, the prediction performance of histopathological-image-only model was better than blood-biomarker-only model. However, in the T3 stage, the biomarker-only model was superior to image-only model (0.685 vs. 0.616, Figure 3(b), Supplemental Table 1).

### 2.3. Performance Evaluation in the External Validation Cohort

**Figure 4:**
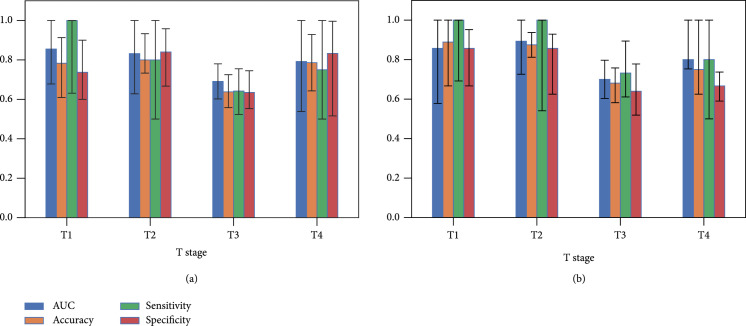
The performance of the MMIL model evaluated in the external validation dataset with different T stages (T1, T2, T3, and T4). (a) The performance of the MMIL model directly tested in external validation cohort. (b) A cohort study, where patients enrolled before 2019 (2013-2018) were utilized for fine-tuning the MMIL model and patients enrolled in 2019 were utilized for the test. The detailed numbers are given in Supplemental Table 2 and Table 3.

MMIL model was further validated in an external validation cohort with 210 subjects from two centres. Our model achieved AUC values of 0.855 (95% CI: 0.678-1.000), 0.832 (95% CI: 0.628-1.000), 0.691 (95% CI: 0.602-0.780), and 0.792 (95% CI: 0.538-1.000) for T1, T2, T3 and T4, respectively (Figure 4(a), Supplemental Table 2), which were slightly inferior compared to the performance in the discovery cohort. Furthermore, we conducted a cohort study, where patients enrolled before 2019 (2013-2018) were utilized for fine-tuning the MMIL model and patients enrolled in 2019 were utilized for the test. Generally, the MMIL model resulted in slightly better AUC compared to the direct test for T1, T3, and T4 with values of 0.857 (95% CI: 0.578-1.000), 0.700 (95% CI: 0.603-0.797), and 0.800 (95% CI: 0.753-1.000), but relative significant performance increase at T2 stage with AUC of 0.893 (95% CI: 0.726-1.000) compared to 0.832 (95% CI: 0.628-1.000) (Figure 4(b), Supplemental Table 3). Simultaneously, we found relative salient sensitivity or specificity increase comparing the performance in the cohort study with the direct test (Supplemental Table 3). These results showed that the model trained with collected data can be employed to predict LNM on future unseen data, indicating its potential in the prospective experiment. And at the same time, the results also suggested that utilizing collected data to fine-tune the MMIL model allowed the model to learn domain bias of each medicine centre to potentially further improve its performance.

### 2.4. Model Interpretation

**Figure 5:**
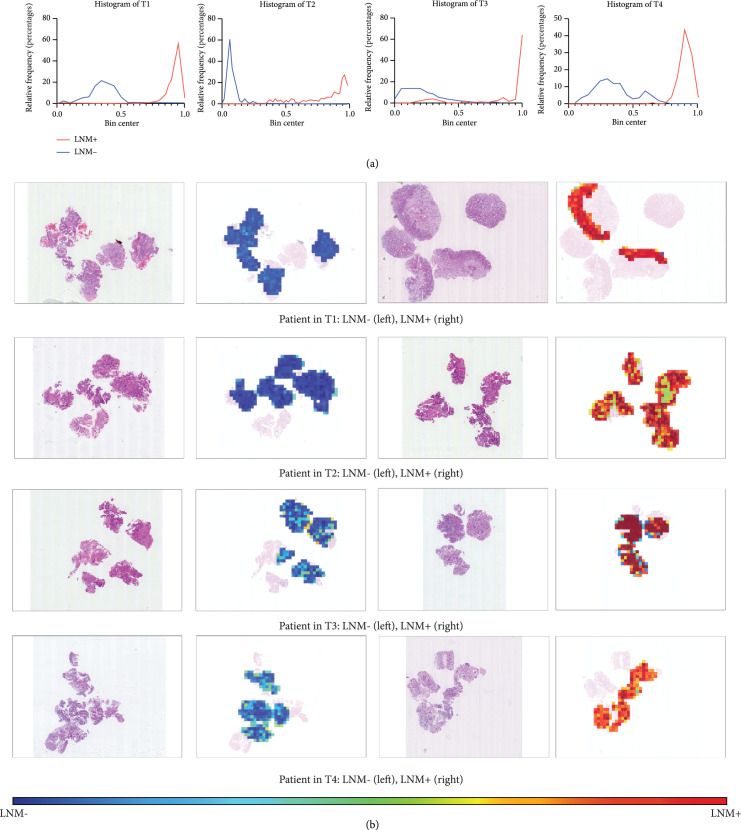
Visualization of the LNM probability of the subregions (tiles) of sample WSIs. (a) The example histograms of the LNM probability scores of WSIs from LNM+ and LNM- patients with different T stages. (b) Examples of LNM probability heatmaps showing the probability distribution on WSIs. The colors reflect LNM probabilities.

To illustrate the decision mechanism of the developed MMIL model, we evaluated the LNM prediction probability distribution in each WSI, where the LNM probability of each subregion (tile) was calculated and visualized. Figure 5 shows the LNM probability histograms of tiles from sample WSIs in the discovery cohort. Those tiles with high LNM probability scores (near 1) and those with low LNM probability scores (near 0) had distinguishable features supporting LNM prediction.

To make it easier for pathologists to understand the characteristics of the tiles with the most importance, we used human-understandable histomic features to characterize these tiles. As shown in Supplemental Figure 1, features such as “Density_distance_for_neighbors_1_means,” “Shape.Solidity_eosin,” “Nucleus.Haralick.SumAverage.Mean_eosin,” and “Size.Area_eosin” represented the histomic features with significant alterations between LNM+ and LNM- WSIs in different T stages. Supplemental Figure 1A shows that the tumor cells of LNM+ patients were denser than those of LNM- patients, which was consistent with the finding of the previous study showing that cell density modulates metastatic aggressiveness in colorectal cancer [21]. Supplemental Figures 1B and 1C shows enhanced texture, abnormal shape, and enlarged size of aggressive tumor cell nuclei in LNM+ patients, indicating increased cell division and increased heterogeneity.

## 3. Discussion

In the present study, we developed and validated a multimodal system that incorporated features of pretreatment digitalized histopathological images and tumor-specific biomarkers for predicting LNM status in patients with CRC. Our model could predict LNM status and attained robust performance exceeding previously reported performance. More importantly, we provided a novel and easy-to-use model based on biopsy-acquired H&E slides and tumor-specific biomarkers that are routinely examined before treatment, making it well suited for routine clinical practice and assisting treatment decision for individualized CRC patient.

Moreover, we demonstrated that MMIL model outperformed each of the individual features alone (digital pathological image or tumor-specific biomarkers). Additionally, we believed there was significant biological rationale for individual component: (1) pretreatment H&E image reflects the tumor cell features and relationship between tumor cell and environment directly, and recent advances in AI have driven digitalized pathology to be a novel and efficient way to solve challenging clinical tasks; (2) pretreatment tumor-related biomarkers had been reported to significantly correlate with LNM and served as important markers for prognosis in patients with CRC [22]. Therefore, we combined histopathological images and tumor-specific biomarkers, including CEA, CA125, CA19-9, and AFP through deep learning to develop an automatically predictive model. Our study showed that the combined model had a higher predictive power compared to image-only model and biomarker-only model (Figure 3). Furthermore, we found that image-only model outperformed the biomarker-only model in most T stages except T3 stage (Figure 3). The potential reason was that most of the biomarkers, i.e., CEA, CA125, and CA19-9 had significant alternations between LNM-/LNM+ patients in T3 stage which could not be observed in other stages (Table 1). Meanwhile, for T3 stage, CA19-9 was the most distinguished biomarker between LNM-/LNM+ patients (P value<0.0001) and made the greatest contribution in biomarker-only classifier (Table 1, Supplemental Figure 2).

Our MMIL model has multiple advantages for the LNM status predication compared to previous methods [23]. Imaging tests and related radiomics were the most common approaches used to predict LNM status [24]. Two meta-analyses reported suboptimal sensitivities and specificities in the range of 55-78% for predicting LNM with MRI [25]. Huang et al. developed a prediction nomogram including radiomic signature, CT-reported LN status, and CEA level, the C-index were 0.736 and 0.778 in internal and external validation cohort, respectively [26]. Different from prior studies, our method automatically learned latent distinguishable features and therefore avoided the effort of making handcrafted features. Besides, different from most existing MIL methods, which mainly rely on local tile information for inference based on the standard multiple instance (SMI) assumption [27], the MMIL method comprehensively extracts information from the whole WSI. Furthermore, we developed our proposed MMIL model using a large dataset, which is beneficial for the learning-based method since the diversity and heterogeneity of the patient data are considered. In addition, the MMIL method was able to integrate the information of different modalities, which was proven to be more accurate than only referring to the information of one modality (Figure 3 and Supplemental Table 1).

Compared with other T stages, the accurate prediction of LNM status for patients in the T1 stage played a more important role in clinical practice, since the treatment solutions for LNM+ and LNM- patients in this stage greatly differ. Interestingly, the MMIL model had the highest performance for stage T1 compared to the other T stages (Figure 3). Besides, we found that compared to the performance of T_all_, substages including T1, T2, T3, and T4 possessed higher AUC during discriminating LNM+ and LNM- [Figure 3(a), AUC: 0.719 (T_all_) vs. 0.926 (T1), 0.878 (T2), 0.809 (T3), and 0.857(T4)]. The potential reason may be that the underlying features were more similar in specific substage and made them easier to generalize.

We evaluated the generalization of the MMIL model in an external validation dataset consisting of subjects from two different centres. Considering that there is a data gap between different centres, directly applying the pretrained AI models trained on the data set of one centre to the data set of other medical centres normally causes performance degradation. The data gap might be staining heterogeneity between different centres due to the protocol differences in H&E staining, different scanning devices, and individual operational variations. However, the performance degradation of the proposed MMIL model in the external validation was limited, which indicated the generalization ability of our model and its ability to learn domain-invariant features (Protocol differences of discovery cohort and external validation cohort are given in the supplement section). In practice, transfer learning by fine-tuning the developed MMIL model on one centre’s own dataset to familiarize the AI model with the specialization of data from this centre is a possible way to further improve the performance [28]. In our cohort study, the fine-tuning technology brings slight improvement to external validation dataset except T2 cohort. One of the possible reasons is that, compared to other T stages, T2 cohort has more difference between the internal dataset and external dataset. On the external cohort, T2 differences are due mainly to the inappropriate histological heterogeneity of several case series and the uneven distribution between groups. Therefore, fine-tuning strategy brings more performance enhancement.

Recently, concerns have been raised regarding the interpretation of deep learning methods. To illustrate the decision mechanism of the established MMIL model, we developed a visualization method to assess the LNM probabilities of the subregions (tiles) of WSIs. Figure 5 shows samples of LNM probabilities heatmaps to provide a better visualization. The features of deep learning are claimed to be not easy to be understood by human. In the present study, we tried to characterize the high-value tiles (tiles with top LNM+ probabilities and LNM- probabilities) with histomic features, which are easier to be understood. Supplemental Figure 1 shows the histomic features with significant alterations between LNM+ and LNM- patients in to*p*-value tiles, which have potential as a signature to divide LNM+ and LNM- patients.

One limitation of this study is that we trained our model on one centre currently. Training the model on datasets of multiple different centres may allow the network to learn domain-invariant features more effectively and enhance its generalization ability. To this end, we are collecting more subjects from other data centres and will evaluate the performance of our model trained on multiple centres after receiving sufficient data from other medical centres. Another limitation is that our study was a retrospective study, and the quality of H&E-stained images was inconsistent across hospitals, which limited the performance improvement of the MMIL model. Satisfactorily, external validation in independent hospitals shows that the prediction model should be reliable, robust, and generalizable in clinical practice. Additionally, we currently focus on triaging patients into two groups: LNM+ vs. LNM-. However, patients with LNM belong to different N stages (without LNM: N0 stage and with LNM: N1 and N2 stages). One valuable future work can be the fine-grained prediction of the N substage as a multiclass classification task. However, a much larger dataset would be necessary.

## 4. Conclusion

In conclusion, we proposed an AI system based on a deep neural network and MIL for predicting LNM status before treatment. The model that integrated digital biopsy images and tumor-specific biomarkers showed superior predictive power compared to individual modality. Our AI system has clinical value to guide preoperative decision-making based on H&E image and tumor-specific biomarkers in CRC patients.

## 5. Method

### 5.1. Whole-Slide Images Preparation, Annotation, and Preprocessing

All slides were made by staining a 3-*μ*m formalin-fixed paraffin-embedded (FFPE) biopsy section with H&E and then digitized using an Aperio (Leica Biosystems, Buffalo Grove, Illinois, USA) AT2 whole-slide scanner. Tumor cells and glands were manually delineated using the ASAP software (version 1.9, https://computationalpathologygroup.github.io/ASAP/) at 20x magnification (0.5 μm/pixel) by two expert pathologists (Dr. Hailing Liu and Dr. Xinjuan Fan) who have been engaged in digestive pathology for at least five years. In this work, the pathologists first manually annotated the cancer regions on each WSI and used them as ROIs for the following processing step. The ROIs were divided into a set of patches with a size of 512×512 pixels. Patches with less than 20% overlap with the ROIs were excluded before further analysis.

### 5.2. Blood Biomarker Preprocessing

It has been reported that biomarker in the blood can indicate LNM in patients [29]; thus, we collected serum tumor-specific biomarkers such as CEA, CA125, CA19-9, and AFP as the input of the biomarker channel of the MMIL model. Considering the lack of some biomarkers in some patients in the discovery cohort and external validation cohort, we performed multivariate imputation via chained equations to impute the missing biomarkers [30]. As the biomarkers are numeric features, the predictive mean matching method was applied for each biomarker. After data imputation, we normalized the biomarkers using min-max normalization. The distribution of these biomarkers in LNM- and LNM+ patients after data imputation is shown in Supplemental Figure 3.

### 5.3. MMIL System

The framework of the proposed MMIL model is illustrated in Figure 2, which consists of two feature extraction channels (i.e., an image-based feature extraction channel and a biomarker-based feature extraction channel) and an information fusion module. In the image-based feature extraction channel, a MIL method was designed to generate a WSI-level representation vector from the histopathological image. In the biomarker-based feature extraction channel, a fully connected feedforward network was developed to generate a molecular-level representation vector. Then, the information fusion module integrated the two obtained representation vectors via a gating-based attention mechanism and tensor fusion [31] to formulate the final distinguishable information including the representation and the conduct of the classification. The tensor fusion modulated the pairwise feature interactions across modalities by taking the Kronecker product of the feature representations, and the gating-based attention mechanism was able to control the expressiveness of each representation. More specifically, the proposed MIL in the image-based feature extraction channel consisted of three steps: instance-level (tile-level) feature extraction, instance-level (tile-level) feature selection, and bag-level (WSI-level) representation generation [32]. The details of the components are illustrated in the remainder of this section. In the formalization of the MIL, each WSI is regarded as a bag and the patches tiled from the WSI are regarded as instances inside the bag. During the training phase, we chose to use the categorical cross-entropy loss, which is defined as

(1)L=−1N∑i=1N∑c=1Cδyi=clogPyi=c,

where N  denotes the number of samples and C  represents the number of categories. The term $\delta \left(y_{i}=c\right)$ is the indicator function of the $i^{th}$ observation belonging to the ${\text{c}}^{th}$ category. $\;P\left(y_{i}=c\right)$ is the predicted probability by the model.

### 5.4. Instance-Level Feature Extractor

In our AI system, we employed the ResNet-18 model [33] as the instance-level feature extractor, which aimed at automatically learning useful features from patches. From another perspective, this component played the role of information compression, and each inputted patch was transformed into a low-dimensional feature space, which facilitated the following classification stage. In this work, we used a pretrained ResNet-18 model (trained on ImageNet) after removing the final fully connected layer to extract distinguishable features.

### 5.5. Feature Selection Strategy

The feature selection procedure chose the most discriminative instance-level features for generating the bag representation. Removing redundant or irrelevant features can also simplify the following learning task. Since, multiple instance learning is a typic weakly supervised learning scheme, we utilized a weakly supervised instance-level feature selection method proposed in our previous work [34] to conduct the feature selection, which is based on histogram [35] and maximum mean discrepancy [36].

### 5.6. WSI-Level Representation Generator

The WSI-level representation generator in our pipeline generates the bag representation by integrating the extracted and selected most discriminative instance-level features. The attention-based deep MIL method [35] was applied, which made the WSI-level representation generator able to adaptively adjust the contribution of each tiled patch for the final decision. After iteratively and incrementally adjusting the attention weights on the feature of each patch during the training phase, the attention-based operator increased the contribution of the instances that were more related to the corresponding bag label and vice versa.

### 5.7. Network Training Setting

We implemented all the components of the MMIL system including ResNet-18 and the attention-based MIL network in the image-based feature extraction channel, the fully connected feedforward network in the biomarker-based feature extraction channel, the gating-based attention mechanism, tensor fusion, and classification-aimed fully connected layer in the information fusion module [35] with Python and PyTorch [37] framework. The Adam optimizer [38] was used to train the MMIL model. To address the class imbalance problem during the bag-level classification stage, we employed the ‘weighted random sampler’ strategy in the PyTorch to prepare each training batch.

### 5.8. Model Interpretability and Feature Visualization

To explore the distribution of biomarkers in patients with or without LNM, we applied hierarchical clustering to visualize the pattern of tumor-specific biomarkers in CRC patients with LNM using the pheatmap package. We also measured the feature importance of each biomarker in the XGBoost classifier by its contribution to the final prediction of LNM.

We assigned the LNM probability score to different tiles as the measure of the usefulness of these tiles for the LNM prediction. Heatmaps were generated by referring to the assigned LNM probability to reflect the determination of each subregion of the WSI. The histomic features such as cellular morphological features corresponding to nucleus size, shape, and texture, and the spatial relationship between nuclei were also extracted to characterize the high-value regions (with top LNM+ or LNM- probability scores) in the WSI.

### 5.9. Statistic Analysis

The ROC curves of the results from cross-validation were calculated and plotted using the *scikit-learn* [39] in Python. The optimal cutoff point of the ROC curves was determined by referring to the Youden Index [40]. The Wilcoxon rank-sum test was used to compare the two paired groups for each clinical information. Three other standard metrics, i.e., sensitivity, specificity, and accuracy, were also employed in this work to illustrate the performance of the AI system, and the confidence intervals were calculated using the bootstrap method. All statistical tests were two sided and P values less than 0.05 were used to indicate statistical significance.

## Data Availability

The data that support the findings of this study are available from the corresponding authors.

## Supplementary Materials

Supplementary Materials Protocol differences of slide preparation. Network architecture. Supplemental Table 1: performance comparison of LNM prediction among different methods evaluated in the discovery cohort. Supplemental Table 2: comparison of the LNM prediction performance between different methods directly tested in external validation cohort. Supplemental Table 3-1: performance of MMIL model in the cohort study on the external validation dataset. Supplemental Table 3-2: data collection year of the external validation cohort. Supplemental Figure 1: the distinguishable histomic features of high-value tiles between LNM+ and LNM- patients. Supplemental Figure 2: hierarchical clustering of biomarkers in CRC patients and feature importance of biomarkers. Supplemental Figure 3: the distribution of biomarkers in CRC patients from discovery cohort before and after imputation.
