# Supplementary material for: Preoperative Prediction of Lymph Node Metastasis in Colorectal Cancer with Deep Learning
Source: BME Front. 2022 Mar 16;2022:9860179. doi: 10.34133/2022/9860179 (PMC10521754; doi:10.34133/2022/9860179)
Supplement: Supplementary Materials — Protocol differences of slide preparation. Network architecture. Supplemental Table 1: performance comparison of LNM prediction among different methods evaluated in the discovery cohort. Supplemental Table 2: comparison of the LNM prediction performance between different methods directly tested in external validation cohort. Supplemental Table 3-1: performance of MMIL model in the cohort study on the external validation dataset. Supplemental Table 3-2: data collection year of the external validation cohort. Supplemental Figure 1: the distinguishable histomic features of high-value tiles between LNM+ and LNM- patients. Supplemental Figure 2: hierarchical clustering of biomarkers in CRC patients and feature importance of biomarkers. Supplemental Figure 3: the distribution of biomarkers in CRC patients from discovery cohort before and after imputation. [file 9860179.f1.docx]

**Supplementary content**

**Protocol Differences of Slide Preparation**

**(The differences are underlined.)**

**The protocol of hematoxylin and eosin (H&E) staining of the Department of Pathology, the Sixth Affiliated Hospital of Sun Yat-sen University**

1. The resection samples are fixed in formalin for more than 24 h.
2. The pathology residents or senior pathologists section the tumor and margins of the sample and submit them to the labeled blocks.
3. The technician put the blocks into the machine (Sakura Dehydrator).

3、Fixation: fixed with 10% neutral buffered formalin for 3 h.

4、Tissue dehydration:

（1）75% ethanol (30℃): 1 h

（2）85% ethanol (30℃): 1 h

（3）95% ethanol (30℃): 1 h

1. Anhydrous ethanol Ⅰ (30 ℃): 1 h
2. Anhydrous ethanol Ⅱ (30 ℃): 1 h
3. Anhydrous ethanol Ⅲ (30 ℃): 1 h
4. Tissue clearing:

（1）xylene Ⅰ (30 ℃): 1 h

（2）xylene Ⅱ (60 ℃): 1 h

6、Tissue infiltration:

（1）paraffin Ⅰ (60 ℃): 30 min

（2）paraffin Ⅱ (60 ℃): 30 min

（3）paraffin Ⅲ (60 ℃): 30 min

（4）paraffin Ⅲ (60 ℃): 20 min

7、Embedding and section: 4 µm

8、Routine H&E staining:

（1）xylene Ⅰ: 5-10 min

（2）xylene Ⅱ: 5-10 min

（3）95% ethanol: 1-3min

（4）95% ethanol: 1-3 min

（5）80% ethanol: 1 min

（6）Distilled water: 1 min

1. Hematoxylin staining: 5-15 min
2. Gently wash the hematoxylin with running water: 1-3 s
3. 1% hydrochloric ethanol: 1-3 s
4. Gentle wash: 10-30 s
5. Wash with running water to return blue: 10-30 s
6. Water flushing: 10-15 min

（13）Eosin staining: 3-10 min

（14）80% ethanol: 1 mim

（15）95% ethanol Ⅰ: 1 min

（16）95% ethanol Ⅱ: 1 min

（17）100% ethanol Ⅰ: 1 min

（18）100% ethanol Ⅱ: 1 min

（19）xylene Ⅰ: 1 min

（20）xylene Ⅱ: 1 min

（21）xylene Ⅲ: 1 min

（22）Seal with neutral gum

**The protocol of hematoxylin and eosin (H&E) staining of the Department of Pathology, the Fourth Affiliated Hospital of Harbin Medical University**

1. The resection samples are fixed in formalin for more than 24 h.
2. The pathology residents or senior pathologists section the tumor and margins of the sample and submit them to the labeled blocks.
3. The technician puts the blocks into the machine (Sakura Dehydrator).

3、Fixation: fixed with 10% neutral buffered formalin for 4 h.

4、Tissue dehydration:

（1）75% ethanol (30℃): 1 h

（2）85% ethanol (30℃): 1 h

（3）95% ethanol (30℃): 1 h

1. Anhydrous ethanol Ⅰ (30 ℃): 1 h
2. Anhydrous ethanol Ⅱ (30 ℃): 2 h
3. Tissue clearing:

（1）xylene Ⅰ (30 ℃): 30 min

（2）xylene Ⅱ (60 ℃): 30 min

（3）xylene Ⅲ (60 ℃): 30 min

6、Tissue infiltration:

（1）paraffin Ⅰ (60 ℃): 1 h

（2）paraffin Ⅱ (60 ℃): 1 h

（3）paraffin Ⅲ (60 ℃): 2 h

7、Embedding and section: 4 µm

8、Routine H&E staining:

（1） xylene Ⅰ: 5-10 min

（2） xylene Ⅱ: 5-10 min

（3） xylene Ⅲ: 5-10 min

（4） 95% ethanol: 1-3 min

（5） 95% ethanol: 1-3 min

（6） 80% ethanol: 1 min

（7）Distilled water: 1 min

1. Hematoxylin staining: 5-15 min
2. Gently wash the hematoxylin with running water: 1-3 s
3. 1% hydrochloric ethanol: 1-3 s
4. Gentle wash: 10-30 s
5. Wash with running water to return blue: 10-30 s
6. Water flushing: 10-15 min

（14）Eosin staining: 3-10 min

（15）80% ethanol: 1 min

（16）95% ethanol Ⅰ: 1 min

（17）95% ethanol Ⅱ: 1 min

（18）100% ethanol Ⅰ: 1 min

（19）100% ethanol Ⅱ: 1 min

（20）xylene Ⅰ: 1 min

（21）xylene Ⅱ: 1 min

（22）xylene Ⅲ: 1 min

（23）Seal with neutral gum

**Network Architecture**

The detailed structure of the deep learning model is illustrated in this section (The structure of instance-level feature extractor, i.e., ResNet-18, is not included for saving space since it is public and well-known).

Assume there are 128 instances in a WSI, and batch size is 1, the input image feature is with the size (1, 128, 256) and the input biomarker feature is with the size (1, 4). The model structure and parameters are as follows:

| Layer (type) | Output Shape | Param # |
| --- | --- | --- |
| Linear-1 | [1, 128] | 32,896 |
| Tanh-2 | [1, 128] | 0 |
| Linear-3 | [1, 1] | 129 |
| Attention_net_feature-4 | [[-1, 256], [-1, 128]] | 0 |
| Linear-5 | [1, 64] | 16,448 |
| ReLU-6 | [1, 64] | 0 |
| Bilinear-7 | [1, 64] | 65,600 |
| Linear-8 | [1, 64] | 4,160 |
| ReLU-9 | [1, 64] | 0 |
| Dropout-10 | [1, 64] | 0 |
| Linear-11 | [1, 4] | 20 |
| ReLU-12 | [1, 4] | 0 |
| Bilinear-13 | [1, 4] | 4,100 |
| Linear-14 | [1, 4] | 20 |
| ReLU-15 | [1, 4] | 0 |
| Dropout-16 | [1, 4] | 0 |
| Dropout-17 | [1, 325] | 0 |
| Linear-18 | [1, 64] | 20,864 |
| ReLU-19 | [1, 64] | 0 |
| Dropout-20 | [1, 64] | 0 |
| Linear-21 | [1, 64] | 4,160 |
| ReLU-22 | [1, 64] | 0 |
| Dropout-23 | [1, 64] | 0 |
| BilinearFusion-24 | [1, 64] | 0 |
| Linear-25 | [1, 1] | 65 |
| Sigmoid-26 | [1, 1] | 0 |

----------------------------------------------------------------

Total params: 148,462

Trainable params: 148,462

Non-trainable params: 0

----------------------------------------------------------------

Input size (MB): 0.50

Forward/backward pass size (MB): 0.26

Params size (MB): 0.57

Estimated Total Size (MB): 1.33

----------------------------------------------------------------

MMIL Architecture: WSI-level Representation Generator (Attention-base MIL):

Attention-base MIL(

(Attention): Sequential(

(0): Linear(in_features=256, out_features=128, bias=True)

(1): Tanh()

(2): Linear(in_features=128, out_features=1, bias=True)

)

(bag_representation): bag_representation(

A = Attention (Input)

A = Transpose(A, 1, 0)

A = Softmax(A, dim=1)

Output = MatrixMultiplication (Attention, Input)

)

)

MMIL Architecture: Information Fusion Module (Fully-connected Feedforward Network in biomarker feature extraction channel is also included [“biomarker_feature_linear_h2”]):

MMIL_information_fusion(

(fusion): BilinearFusion(

(image_featrue_linear_h1): Sequential(

(0): Linear(in_features=256, out_features=64, bias=True)

(1): ReLU()

)

(image_featrue_linear_z1): Bilinear(in1_features=256, in2_features=4, out_features=64, bias=True)

(image_featrue_linear_o1): Sequential(

(0): Linear(in_features=64, out_features=64, bias=True)

(1): ReLU()

(2): Dropout(p=0.25, inplace=False)

)

(biomarker_feature_linear_h2): Sequential(

(0): Linear(in_features=4, out_features=4, bias=True)

(1): ReLU()

)

(biomarker_feature_linear_z2): Bilinear(in1_features=256, in2_features=4, out_features=4, bias=True)

(biomarker_feature_linear_o2): Sequential(

(0): Linear(in_features=4, out_features=4, bias=True)

(1): ReLU()

(2): Dropout(p=0.25, inplace=False)

)

(post_fusion_dropout): Dropout(p=0.25, inplace=False)

(fusion_feature_encoder1): Sequential(

(0): Linear(in_features=325, out_features=64, bias=True)

(1): ReLU()

(2): Dropout(p=0.25, inplace=False)

)

(fusion_feature_encoder2): Sequential(

(0): Linear(in_features=64, out_features=64, bias=True)

(1): ReLU()

(2): Dropout(p=0.25, inplace=False)

)

)

(classifier): Sequential(

(0): Linear(in_features=64, out_features=1, bias=True)

)

(activation): Sigmoid()

)

**Supplemental Table 1:** Performance comparison of LNM prediction among different methods evaluated in the discovery cohort. T_all_: patients mixed with different T stages

|  | T1 | T2 | T3 | T4 | T_all_ |
| --- | --- | --- | --- | --- | --- |
| Images and Biomarkers |  |  |  |  |  |
| AUC | 0.926 (0.864-0.988) | 0.878 (0.824-0.933) | 0.809 (0.775-0.843) | 0.857 (0.799-0.915) | 0.719 (0.694-0.744) |
| Sensitivity | 88.9% (66.7%-100%) | 96.8% (90.0%-100%) | 83.0% (78.2%-88.1%) | 93.3% (87.0%-97.8%) | 69.7% (66.2%-73.2%) |
| Specificity | 91.1% (81.8%-96.7%) | 77.9% (69.9%-86.0%) | 75.6% (72.0%-79.6%) | 75.6% (66.7%-84.8%) | 71.6% (68.9%-74.4%) |
| Accuracy | 90.8% (83.1%-98.5%) | 81.1% (74.4%-87.2%) | 78.1% (74.7%-81.2%) | 84.9% (78.5%-89.5%) | 70.9% (68.8%-73.0%) |
| Images Only |  |  |  |  |  |
| AUC | 0.850 (0.734-0.966) | 0.701 (0.598-0.803) | 0.616 (0.578-0.654) | 0.763 (0.676-0.851) | 0.632 (0.559-0.664) |
| Sensitivity | 77.8% (54.5%-100%) | 72.0% (55.0%-92.0%) | 58.0% (52.8%-62.9%) | 72.1% (61.8%-82.8%) | 53.7% (48.8%-58.4%) |
| Specificity | 82.0% (71.7%-93.3%) | 66.7% (58.8%-77.3%) | 67.0% (63.3%-70.8%) | 84.6% (75.0%-94.0%) | 71.1% (67.8%-74.6%) |
| Accuracy | 81.4% (72.9%-89.8%) | 67.7% (59.7%-74.2%) | 63.4% (60.4%-66.5%) | 77.5% (70.0%-84.2%) | 64.4% (61.8%-66.9%) |
| Biomarkers Only |  |  |  |  |  |
| AUC | 0.563 (0.278-0.847) | 0.672 (0.540-0.803) | 0.685 (0.653-0.717) | 0.756 (0.666-0.845) | 0.682 (0.651-0.712) |
| Sensitivity | 33.3% (0.0%-66.7%) | 64.5% (46.4%-81.2%) | 68.6% (64.5%-72.7%) | 64.4% (55.3%-75.0%) | 55.8% (52.0%-60.0%) |
| Specificity | 89.3% (81.1%-96.3%) | 78.4% (73.4%-85.0%) | 78.7% (75.8%-81.6%) | 78.0% (68.7%-87.1%) | 77.1% (74.6%-79.7%) |
| Accuracy | 81.5% (73.8%-89.2%) | 76.1% (69.6%-82.1%) | 74.9% (72.3%-77.3%) | 70.9% (63.4%-76.2%) | 69.4% (67.1%-71.7%) |
| Radiology Image (CT) |  |  |  |  |  |
| Sensitivity | 77.8% (44.4%-88.9%) | 64.5% (48.4%-77.4%) | 66.7% (62.6%-70.0%) | 64.4% (54.4%-72.2%) | 67.8% (63.8%-71.3%) |
| Specificity | 85.7% (76.8%-91.1%) | 70.6% (62.1%-77.1%) | 44.7% (41.8%-47.7%) | 50.0% (37.8%-58.5%) | 51.2% (48.0%-54.1%) |
| Accuracy | 84.6% (75.4%-90.8%) | 69.6% (62.0%-75.0%) | 53.0% (50.7%-55.0%) | 57.6% (51.2%-66.9%) | 57.2% (54.6%-59.4%) |

**Supplemental Table 2:** Comparison of the LNM prediction performance between different methods directly tested in external validation cohort.

|  | T1 | T2 | T3 | T4 |
| --- | --- | --- | --- | --- |
| Images and Biomarkers |  |  |  |  |
| AUC | 0.855 (0.678-1.000) | 0.832 (0.628-1.000) | 0.691 (0.602-0.780) | 0.792 (0.538-1.000) |
| Sensitivity | 100.0% (63.1%-100.0%) | 80.0% (50.0%-100.0%) | 64.2% (52.3%-75.5%) | 75.0% (50.0%-100.0%) |
| Specificity | 73.7% (60.0%-90.0%) | 84.0% (66.7%-95.8%) | 63.5% (55.3%-74.5%) | 83.3% (51.6%-99.6%) |
| Accuracy | 78.3% (60.9%-91.3%) | 80.0% (73.3%-93.3%) | 63.8% (55.8%-72.5%) | 78.6% (64.3%-92.9%) |
| Images Only |  |  |  |  |
| AUC | 0.776 (0.508-1.000) | 0.760 (0.507-1.000) | 0.577 (0.480-0.674) | 0.729 (0.378-1.000) |
| Sensitivity | 75.0% (25.0%-100.0%) | 80.0% (28.4%-99.5%) | 75.5% (64.2%-84.9%) | 75.0% (50.0%-87.5%) |
| Specificity | 89.5% (76.5%-100.0%) | 80.0% (62.5%-92.0%) | 40.0% (29.4%-48.2%) | 83.3% (50.0%-83.3%) |
| Accuracy | 87.0% (78.3%-95.7%) | 80.0% (70.0%-93.3%) | 53.6% (44.2%-60.9%) | 78.6% (50.0%-92.9%) |
| Biomarkers Only |  |  |  |  |
| AUC | 0.296 (0.100-0.496) | 0.576 (0.331-0.821) | 0.550 (0.465-0.636) | 0.500 (0.150-0.850) |
| Sensitivity | 0.0% (0.0%-0.0%) | 60.0% (20.0%-80.0%) | 56.6% (41.7%-69.0%) | 25.0% (12.5%-37.5%) |
| Specificity | 84.2% (76.2%-94.1%) | 64.0% (44.0%-76.0%) | 67.1% (58.0%-79.0%) | 83.3% (50.0%-83.3%) |
| Accuracy | 69.6% (56.5%-82.6%) | 63.3% (46.7%-76.7%) | 61.6% (61.6%-61.6%) | 50.0% (35.7%-64.3%) |

**Supplemental Table 3-1:** Performance of MMIL model in the cohort study on the external validation dataset. Patients enrolled before 2019 (2013-2018) were utilized for finetuning the MMIL model and patients enrolled in 2019 were utilized for the test.

|  | T1 | T2 | T3 | T4 |
| --- | --- | --- | --- | --- |
| Cohort Study |  |  |  |  |
| AUC | 0.857 (0.578-1.000) | 0.893 (0.726-1.000) | 0.700 (0.603-0.797) | 0.800 (0.753-1.000) |
| Sensitivity | 100.0% (69.2%-100.0%) | 100.0% (54.1%-100.0%) | 73.2% (61.1%-89.4%) | 80.0% (50.0%-100.0%) |
| Specificity | 85.7% (66.7%-95.2%) | 85.7% (62.5%-92.9%) | 64.0% (51.9%-77.8%) | 66.7% (59.0%-73.7%) |
| Accuracy | 88.9% (66.7%-100.0%) | 87.5% (81.2%-93.7%) | 68.1% (58.2%-75.8%) | 75.0% (62.5%-100.0%) |

**Supplemental Table 3-2:** Data collection year of the external validation cohort

| Year | T1 | T2 | T3 | T4 | Sum |
| --- | --- | --- | --- | --- | --- |
| 2013 | 1 | 0 | 0 | 0 | 1 |
| 2014 | 1 | 0 | 0 | 0 | 1 |
| 2016 | 2 | 0 | 0 | 0 | 2 |
| 2017 | 5 | 1 | 4 | 0 | 10 |
| 2018 | 5 | 14 | 45 | 6 | 70 |
| 2019 | 10 | 16 | 92 | 8 | 126 |
| Sum | 24 | 31 | 141 | 14 | 210 |

Patients enrolled before 2019 (2013-2018) were utilized for finetuning the MMIL model and patients enrolled in 2019 were utilized for the test. The finetuning data accounted for 58.3% (T1), 48.4% (T2), 34.8% (T3), 42.9% (T4), and 40.0% (T_all_) of the patients at each stage.


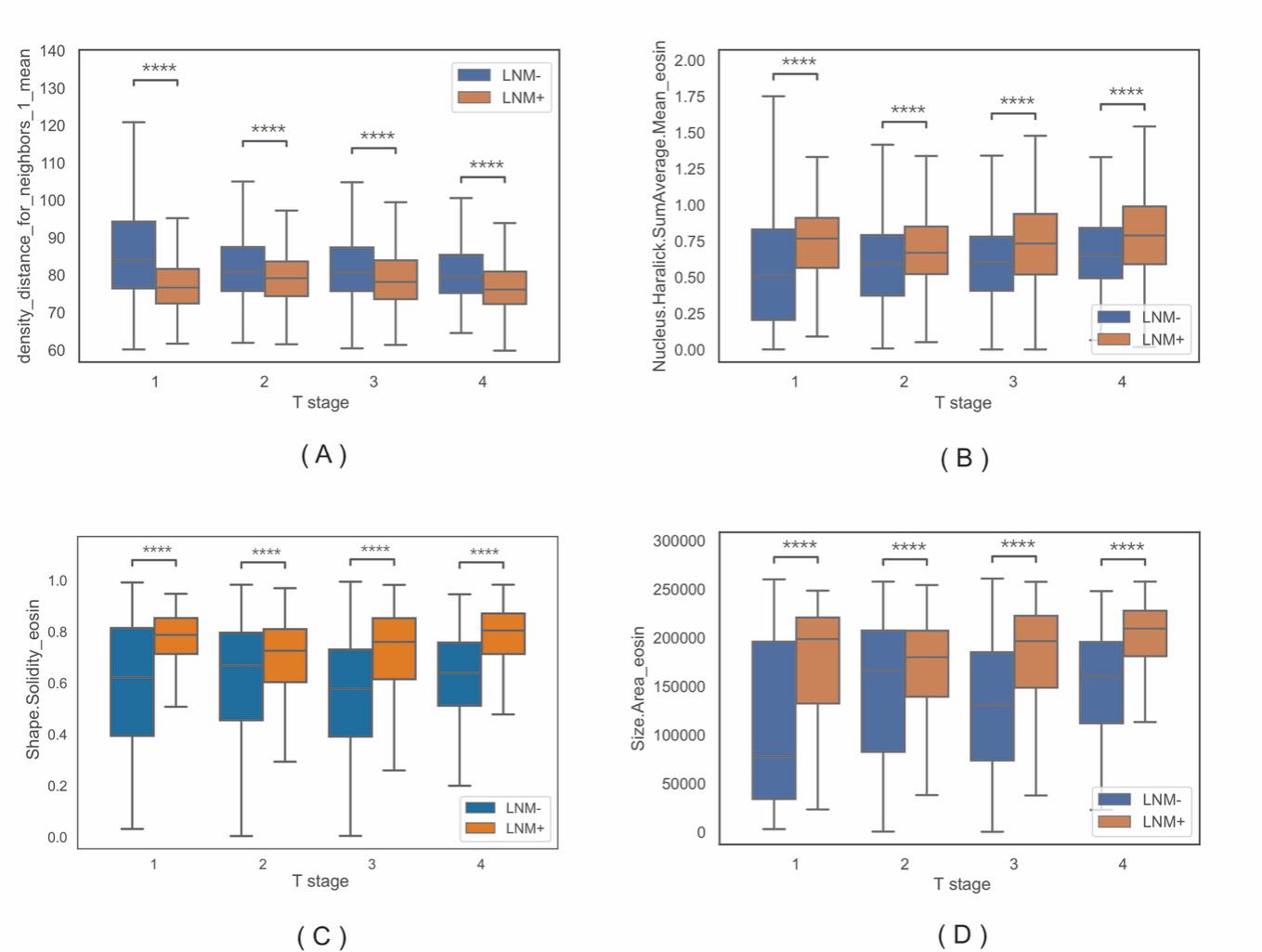


**Supplemental Figure 1:** The distinguishable histomics features of high-value tiles between LNM+ and LNM- patients. **(A)** density_distance_for_neighbors_1_means, **(B)** Nucleus.Haralick.SumAverage.Mean_eosin, **(C)** Shape.Solidity_eosin, (D) Size.Area_eosin.


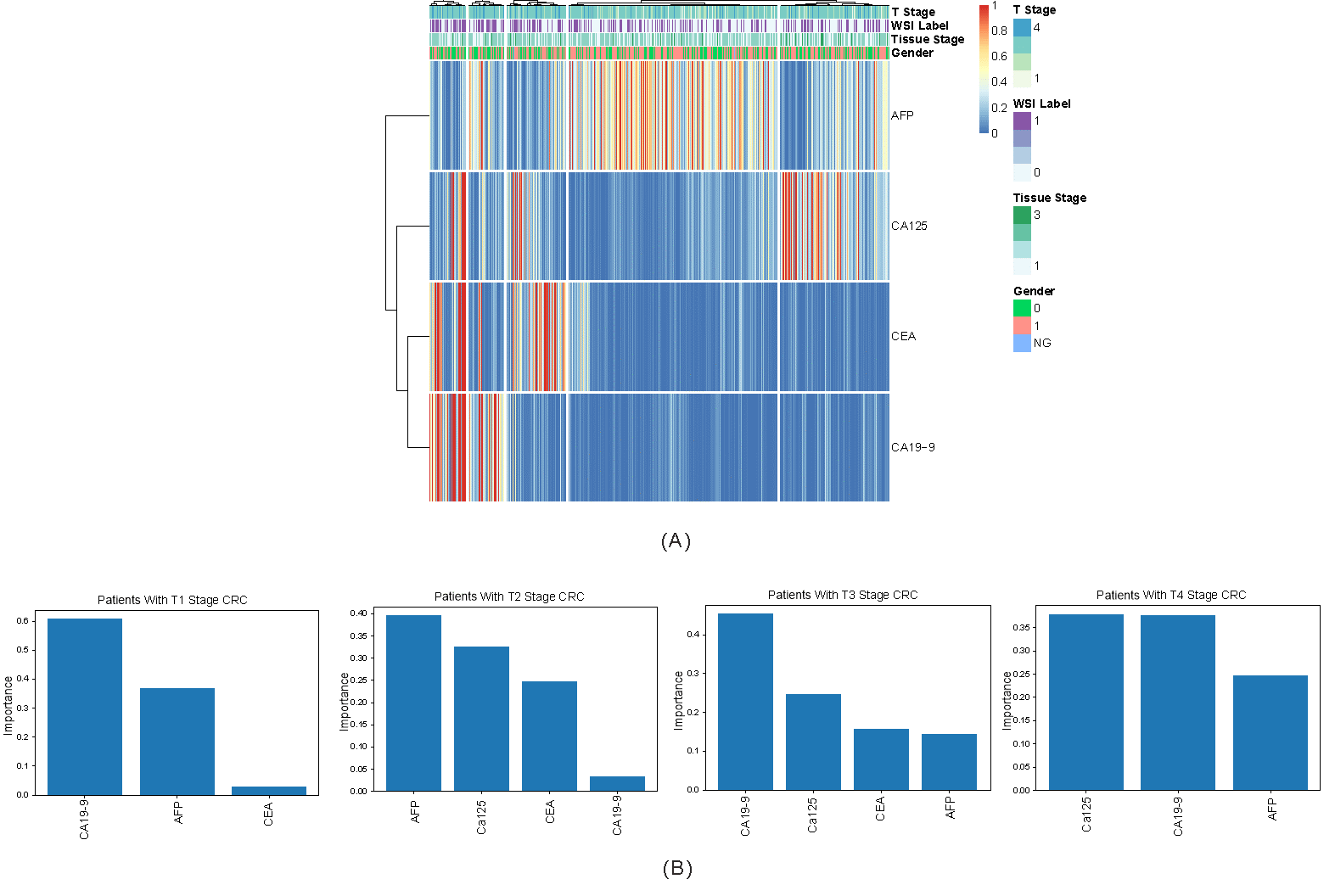


**Supplemental Figure 2: (A)** Hierarchical clustering of biomarkers (CEA, CA125, CA19-9, AFP) in CRC patients from discovery cohort after imputation and normalization. **(B)** Feature importance of biomarkers in XGBoost classifier distinguishing LNM status for patients with stage T1, T2, T3, and T4 CRC**.**


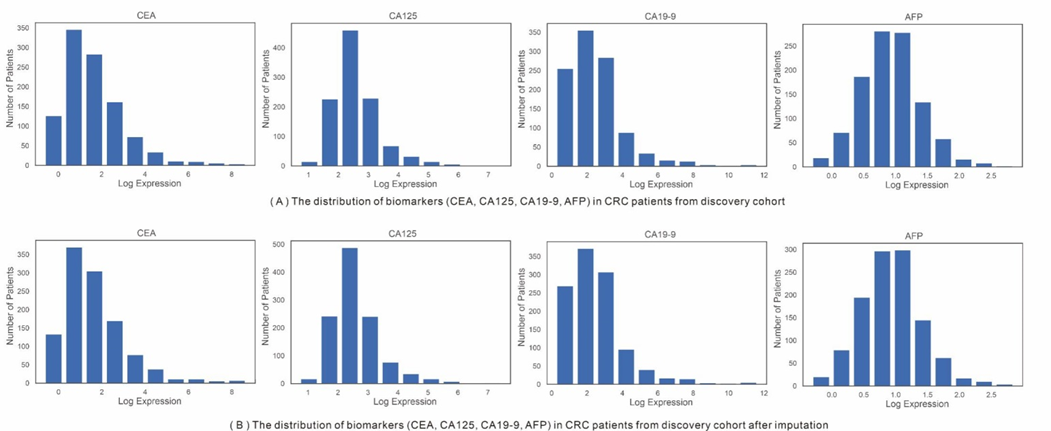


**Supplemental Figure 3:** The distribution of biomarkers in CRC patients from discovery cohort before and after imputation. **(A)** The distribution of biomarkers including CEA, CA125, CA19-9, and AFP in CRC patients for discovery cohort. **(B)** The distribution of biomarkers including CEA, CA125, CA19-9, and AFP in CRC patients for discovery cohort after imputation
